# Gelatin-Mediated Interfacial Engineering of Cellulose-Based Phase Change Foams for Temperature-Adaptive Thermal Regulation

**DOI:** 10.34133/research.1473

**Published:** 2026-11-04

**Authors:** Ya Zhou, Yingfeng Zuo, Huan Gu, Ping Li, Xiaolong Guo, Yiqiang Wu

**Affiliations:** State Key Laboratory of Utilization of Woody Oil Resource, School of Materials and Energy, Central South University of Forestry and Technology, Changsha, Hunan, China.

## Abstract

Thermally insulating materials are used for thermal management in various applications, but conventional materials only block heat transfer, leading to a substantial trade-off between preventing external heat penetration and retaining indoor heat. Here, we report a temperature-adaptive bio-composite foam (CPF) constructed via gelatin-mediated interfacial engineering. The material features an isotropic hierarchical porous structure with a maximum porosity of 95.9%, exhibiting a thermal conductivity of 0.039 W/(m·K) and a noise reduction coefficient (NRC) of 0.54. Benefiting from the anchoring effect of gelatin on phase-change microcapsules (PCMCs) at the plateau borders, the foam withstands a load up to 481 times its own weight without deformation. It also possesses an enthalpy of 140.7 J/g, effectively reconciling the trade-off between thermal insulation and heat dissipation in enclosed spaces. Moreover, the reversible molecular-scale structural design endows the CPF with closed-loop recyclability. This study provides a promising strategy for the development of all-weather, temperature-adaptive thermal management materials.

## Introduction

As the greenhouse effect intensifies and extreme weather events become more common, energy consumption in the building and construction sectors accounts for an increasing proportion of global energy usage. The energy required for heating and cooling alone exceeds 10% of the global total [1–5]. As global carbon reduction goals advance, it is becoming increasingly necessary to strengthen thermal management to save energy and improve energy efficiency [6–10]. Currently, building insulation materials are primarily composed of polymer foams, such as polystyrene (PS), polypropylene (PP), polyurethane (PU), and expanded polyethylene (EPE) [11]. However, most of these materials are derived from nonrenewable resources, and their production processes consume high amounts of energy and have a large carbon footprint. The materials are also nonbiodegradable [12–16].

Cellulose is the most abundant biopolymer on the planet and is characterized by high mechanical strength, sustainability, and biodegradability, making it an excellent candidate for developing lightweight bio-foam [17,18]. Converting cellulose into low-density, low-thermal-conductivity porous materials typically involves complex mechanical defibration or alkali–urea solution regeneration processes [19–21]. Other strategies use toxic chemical surfactants [such as sodium dodecyl sulfate (SDS)] [22,23] and cross-linking agents (such as glutaraldehyde and borates) to produce lightweight structures with enhanced mechanical properties [24–26]. Recent studies have demonstrated that hierarchical ceramic aerogels constructed from cellulose-derived microcrumpled structures exhibit excellent thermal insulation, mechanical stability, and adaptability to extreme environments, offering a new materials design strategy for multifunctional thermal management in extreme environments such as aerospace applications [27]. However, similar to conventional polymer foams, these aerogels primarily rely on the low thermal conductivity of stagnant air to achieve thermal insulation, yet lack active heat-conversion and thermal-regulation mechanisms. Consequently, they face an inherent trade-off between suppressing external heat ingress and dissipating internal heat [28–31]. Developing temperature-adaptive thermal management materials that enable dynamic temperature response and regulation can help cope with dynamic environmental conditions.

Phase-change materials (PCMs) have temperature-responsive properties that allow them to store and release latent heat through reversible phase transitions under near-constant temperatures. This provides thermal regulation for adaptive smart thermal management and advanced energy-saving systems [32–34]. Recent studies have reported many temperature-adaptive composite materials based on PCMs, including infrared stealth [35–37], dynamic radiative cooling [38,39], and lignocellulosic thermostatic materials [30]. However, these functional integrations typically require multi-step encapsulation processes or multilayer structural designs. Weak interfacial interactions between PCMs and scaffold materials may compromise the long-term durability, thereby limiting the practical applications of the resulting materials [40].

In this work, we employed a one-step molding strategy to obtain a gelatin-mediated, interface-engineered cellulose-based composite phase change foam to achieve temperature adaptability and thermal regulation to maintain indoor thermal comfort (Fig. 1). In the bio-composite foam (CPF), cellulose served as the substrate, while the introduction of phase-change microcapsules (PCMCs) endowed the material with dynamic thermal management properties. By using gelatin as both a foaming agent and an interfacial binder, the PCMC particles were in situ anchored, which substantially enhanced the structural stability and thermal energy management efficiency of the material (Fig. 1A). In steady-state scenarios, the entangled network of cellulose and gelatin molecular chains and the hierarchical porous structure endowed the CPF with a porosity of 95.9% and a thermal conductivity of 0.039 W/(m·K) (Fig. 1C). Under transient temperature fluctuations, the CPF absorbed and released heat via the PCMC phase transition, providing an enthalpy of 140.7 J/g, which enhanced the heat capacity of the CPF and delayed or prevented heat propagation. The CPF specimen with a thickness of 15 mm demonstrated exceptional sound absorption capabilities, with a noise reduction coefficient (NRC) of 0.54. Compared with traditional commercial foams (EPE and PS foams), CPF possesses moldability, thermal stability, closed-loop recyclability (Fig. 1D). Overall, this multifunctional, sustainable CPF shows potential for next-generation thermal management and acoustic protection applications.

**Fig. 1.**
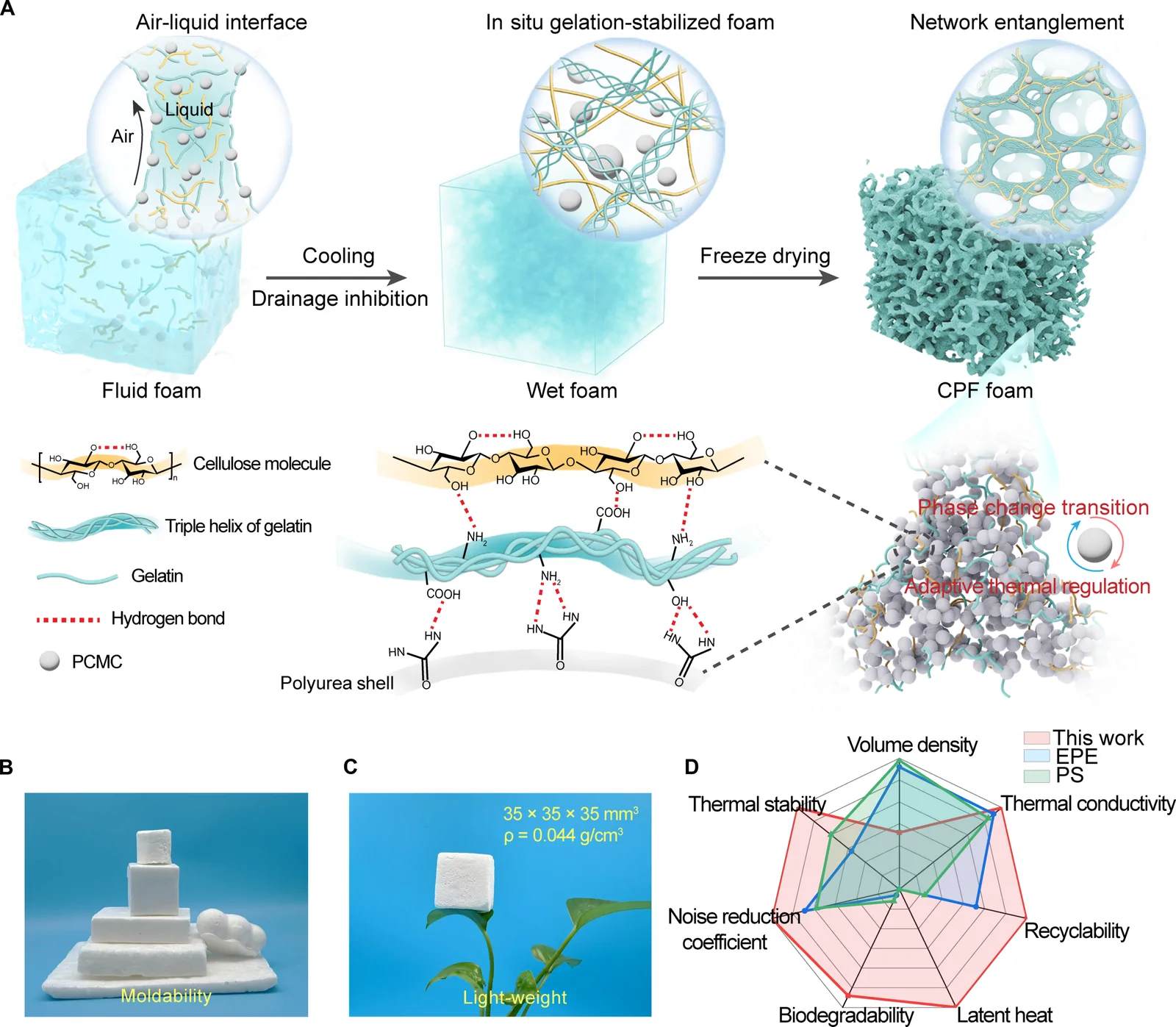
Temperature-adaptive CPF. (A) Preparation process and structural design of CPF. (B) Digital photographs of CPF molded into various shapes. (C) Digital photograph of CPF3.5 placed on a green radish leaf. (D) Comparison of CPF and commercial foams in terms of density, thermal conductivity, recyclability, latent heat, biodegradability, noise reduction coefficient, and thermal stability.

## Results and Discussion

### Interfacial structure design and characterization of CPF

The structural integrity and thermal adaptivity of the cellulose-based foams are determined by the stability of the precursor wet foams and the distribution uniformity of encapsulated particles. CPFs were fabricated via a mechanical frothing process and subsequent freeze-drying. In this process, cellulose dispersions were blended with PCMCs at different mass ratios, followed by the addition of a small amount of ethyl acetate (EA) to facilitate emulsification. Subsequently, a gelatin solution was introduced, and the resulting mixture was subjected to high-shear stirring to generate stable wet foams. Notably, gelatin is a natural amphiphilic biomacromolecule that possesses unique interfacial activity and thermoreversible gelation behavior [41]. At the air–water interface, the hydrophobic and hydrophilic groups along the gelatin molecular chains enable rapid adsorption and reduction of surface tension, thereby stabilizing the bubble lamellae. To further solidify the porous structure, the wet foam was pre-frozen at −18 °C. During the cooling process, the system underwent 2 concurrent physical transformations. On the one hand, when the temperature dropped below the gelation point, gelatin underwent a conformational transition from disordered molecular chains to triple-helical structures, followed by gelation. This process enabled the in situ immobilization of the bubbles and effectively suppressed liquid drainage and bubble coalescence in the wet foam (Fig. 2A). On the other hand, the free water within the system rapidly crystallized into ice crystals at low temperature, which, together with the gelatin network, served as a template for constructing a solid foam gel containing both air and ice templates. Finally, during freeze-drying, the ice crystals were sublimated in situ, leaving behind a hierarchical porous microstructure in the resulting CPF. To validate this stabilization mechanism, we investigated the influence of different foaming agents [polyvinyl alcohol (PVA) and SDS] on the foam stability. Although PVA and SDS exhibited comparable foaming capacities to gelatin, the CPF0 wet foam demonstrated higher viscosity and superior long-term morphological stability (Fig. S1). Rheological measurements (Fig. 2B) confirmed that a sol–gel transition occurred in CPF0 during cooling, in which the in situ-formed viscoelastic network immobilized bubbles. This suppressed Ostwald ripening and mitigated the detrimental effects of interfacial tension on foam pore size. Optical microscopy was employed to monitor changes in wet foams with different PCMC concentrations over time. The bubble growth rates remained consistently low, indicating that bubble coalescence was inhibited (Fig. S2). As water evaporated from the wet foams, interfacial regions of adjacent bubbles fused, leaving PCMCs dispersed within the foam (Fig. S3). Scanning electron microscopy (SEM) images revealed that the dried CPF possessed a continuous hierarchical porous structure. Upon increasing the PCMC content, the plateau borders gradually thickened, within which PCMCs were anchored by the physically entangled network of cellulose and gelatin chains (Fig. 2C and D and Fig. S4). The surface of each PCMC was uniformly encapsulated by a gelatin layer to form a continuous interfacial phase that promoted integration between PCMCs and the matrix. Furthermore, energy-dispersive x-ray spectroscopy (EDS) element maps (Fig. S5) confirmed a homogeneous distribution of C, O, and N throughout the foam skeleton.

**Fig. 2.**
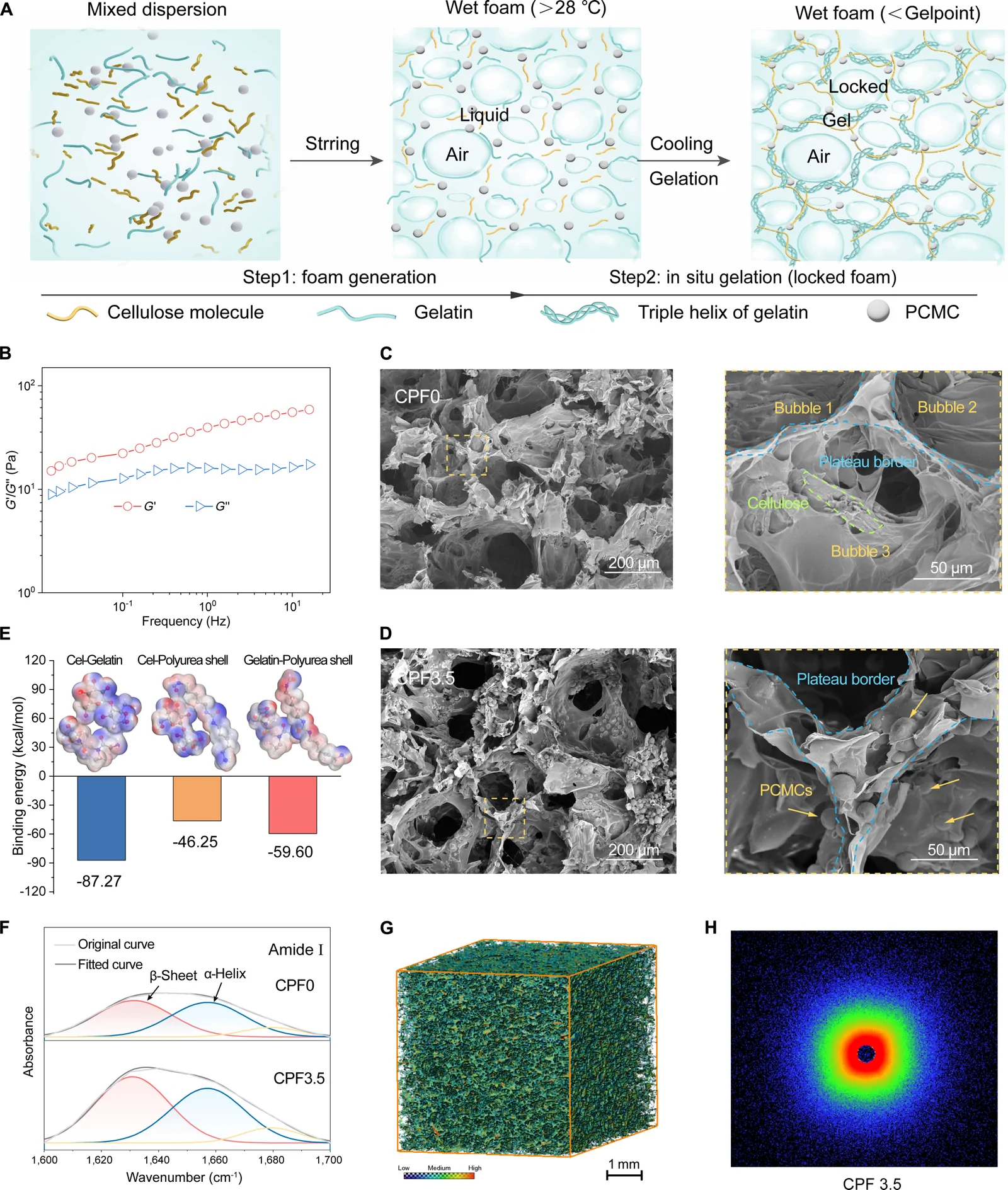
Formation and characterization of CPF. (A) Diagram showing the formation of CPF structure. (B) Frequency-dependent modulus curves of CPF3.5 wet foam. (C) SEM image of CPF0 and (D) CPF3.5 after freeze-drying. (E) ESP maps and BE for different interaction pairs: cellulose-gelatin, cellulose-polyurea, and gelatin-polyurea. (F) Deconvolution of the amide I band in the FT-IR spectra of CPF0 and CPF3.5. (G) Skeleton density distribution maps of CPF3.5 based on micro-CT reconstruction. (H) 2D SAXS pattern of CPF3.5, illustrating its microstructure and interfaces.

To investigate interactions between cellulose, gelatin, and PCMCs within the CPF, we calculated the electrostatic potential (ESP) and binding energy (BE) of the components using density functional theory (DFT) (Fig. 2E). The BE between gelatin and cellulose was −87.27 kcal/mol, indicating the formation of a robust skeletal network via multiple hydrogen bonds. The magnitude of the BE between gelatin and polyurea was greater than that between cellulose and polyurea, demonstrating that gelatin acted as an interfacial adhesive that enhanced the binding strength of PCMCs. Thus, it facilitated their stable anchoring onto the cellulose matrix and prevented their agglomeration or detachment. To further investigate these interfacial interactions at the molecular scale, Fourier transform infrared (FT-IR) spectroscopy was conducted (Fig. S6). The FT-IR spectra of the CPF exhibited the characteristic C–O–C stretching vibrations of cellulose alongside the amide I, II, and III bands of gelatin and PCMCs, confirming the molecular-scale integration of the 3 components [42–44]. The O–H stretching vibration peak of cellulose at 3,333 cm^−1^ exhibited a red shift after the incorporation of gelatin and PCMCs, indicating the formation of hydrogen-bonding interactions among the components [45–47]. Deconvolution of the amide I band (Fig. 2F and Fig. S7) revealed that, compared with CPF0, sample CPF3.5 exhibited a larger β-sheet peak area, suggesting that the incorporation of PCMCs induced a conformational rearrangement of the gelatin molecular chains [48,49]. This rearrangement reinforced intermolecular hydrogen bonds and established a continuous interfacial phase between the PCMCs and the cellulose/gelatin network. Collectively, these results demonstrate that the gelatin-mediated interface firmly anchors PCMCs within the matrix via synergistic hydrogen bonding and physical entanglement, rather than simple physical blending, thereby highlighting the critical role of gelatin in constructing the CPF system. The microstructure of CPF3.5 was characterized using micro-computed tomography (CT) and 2-dimensional (2D) small-angle x-ray scattering (SAXS). The 3D reconstructed models derived from cross-sectional images (Fig. 2G and Fig. S8) revealed an isotropic hierarchical porous structure. The reconstructed 3D network also exhibited a uniform spatial distribution, confirming the homogeneous distribution of PCMCs within the matrix. The concentric diffraction rings in the 2D SAXS patterns provided additional evidence for the high degree of isotropy and structural uniformity of the foam at the nanoscale.

### Mechanical and thermal insulation properties

The skeletal and pore structures of the foam play crucial roles in determining its mechanical properties and thermal conductivity. Figure 3A illustrates the load-bearing capacity and structural stability of CPF3.5, which could support 481 times its own weight without deformation. The compressive performance of CPF3.5 was also evaluated (Fig. 3B), and its stress–strain curve exhibited 3 distinct stages: elastic deformation, plastic deformation, and densification. At a compressive strain of 50%, the compressive stress of CPF3.5 was 86.9 ± 1.8 kPa (*n* = 3). CPF3.5 exhibited favorable flexural toughness (Fig. 3C and Fig. S10), with a fracture strain reaching 50% to 70% and a flexural strength of 69.3 ± 6.7 kPa (*n* = 3). After reaching the maximum stress, the stress began to decline, indicating the foam’s ability to absorb energy. These results suggest that the support strength and flexural toughness of CPF3.5 were due to the physically entangled network of cellulose/gelatin molecular chains, interfacial hydrogen bonding, and the stable anchoring of PCMCs. Collectively, these factors enhanced the deformation resistance and energy dissipation capabilities of CPF3.5.

**Fig. 3.**
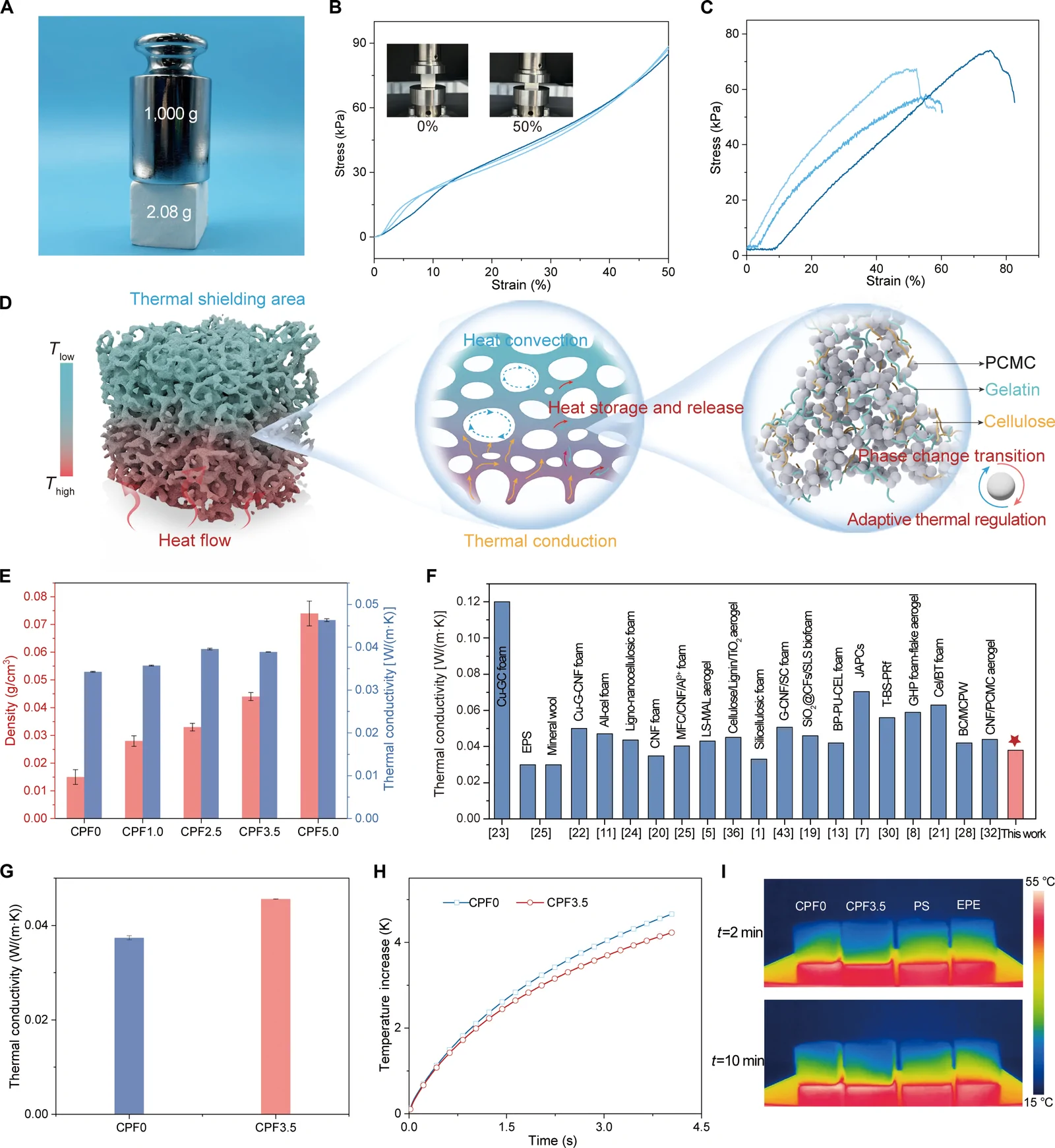
Thermal insulation properties of CPF. (A) Digital photograph of CPF3.5 supporting 481 times its own weight. (B) Compressive stress–strain curve and digital photos during the compression test of CPF3.5. (C) Three-point bending stress–strain curves of CPF3.5. (D) Schematic showing the thermal insulation mechanism of the CPF. (E) Thermal conductivity of CPF with different microcapsule contents. Data are presented as mean ± standard deviation (*n* = 3). (F) Comparison of thermal conductivity between CPF3.5 and previously reported insulating foams. (G) Thermal conductivity of CPF0 and CPF3.5 at 50 °C. (H) Temperature rise curves of CPF0 and CPF3.5 during thermal conductivity testing at 50 °C. (I) Thermal infrared images of CPF0, CPF3.5, and commercial foams (EPE and PS) on a hot plate set to 50 °C.

The thermal conductivity is a critical indicator for evaluating the thermal insulation performance of foams. As shown in Fig. 3E, when the ratio of cellulose to PCMCs in the CPF is increased from 1:0 to 1:5, the thermal conductivity increases from 0.035 W/(m·K) to 0.046 W/(m·K), and these values are comparable to those of conventional thermal insulation materials (Fig. 3F). Notably, the thermal conductivity of CPF is comprehensively influenced by both the pore structure and porosity. With the increasing content of PCMCs, the plateau borders of CPF0 to CPF5 gradually thicken and the skeleton density increases, which increases the solid conduction paths of heat within the CPFs, resulting in a higher thermal conductivity. We further measured the thermal conductivity of CPF0 and CPF3.5 at 50 °C (Fig. 3G). The thermal conductivity of CPF3.5 [0.046 W/(m·K)] is higher than that of CPF0 [0.037 W/(m·K)], consistently highlighting this characteristic. Nevertheless, compared with conventional thermal insulation materials, the CPF exhibits its unique advantages. By comparing the temperature rise curves during thermal conductivity testing (Fig. 3H and Fig. S11), it is found that the temperature rise of CPF3.5 is lower than that of CPF0, which is attributed to the endothermic effect of PCMCs that effectively reduces the thermal diffusion rate and helps to delay heat propagation. To further illustrate the thermal delay characteristic of CPF3.5, a thermal infrared camera was employed to capture the infrared thermographs of CPF0, CPF3.5, and commercial foams (EPE and PS) on a hot plate at 50 °C (Fig. 3I). It is clearly observed that, within the same duration, the heat propagation rate of CPF3.5 is markedly lower than that of the other materials. This result confirms that CPF foam exhibits more stable thermal insulation performance than conventional thermal insulation materials under fluctuating temperature conditions.

### Thermal regulation performance and thermal stability

The latent heat of PCMCs is the key factor determining the thermal regulation capability of CPF (Fig. 4A). To further evaluate the phase change behavior of CPF during melting and crystallization, differential scanning calorimetry (DSC) testing was performed on the samples. The CPFs with different PCMC loadings exhibit similar DSC curve trends, and their phase change enthalpies (Δ*H*) increase as a function of the PCMC content, and enthalpy of phase transition increased with increasing PCMC content (Fig. 4B and Fig. S12). When the mass ratio of cellulose to PCMCs was 1:5, the CPF exhibited the highest enthalpy values, with melting (*ΔHm*) and crystallization enthalpies (*ΔHc*) of 157.3 J/g and 161.2 J/g, respectively, indicating its excellent latent heat storage capability (Table S1). Notably, the amount of PCMCs added has almost no effect on the phase transition temperatures of CPF. The crystallization temperature (*T*_c_) and melting temperature (*T*_m_) are 21.0 and 36.5 °C, respectively, which are highly consistent with those of PCMCs (Fig. 4C). Considering the chemical composition, the phase-change capability of CPF originates from n-eicosane in PCMCs. The encapsulation by polyurea restricts the mobility of the n-eicosane molecular chains, leading to reductions in crystallinity, enthalpy, and phase change temperatures. This is consistent with the x-ray diffraction (XRD) results (Fig. 4D), where the diffraction peaks of the CPF are similar to those of n-eicosane. Meanwhile, this also indicates that the cross-linked network of fibers and gelatin exerts no influence on the crystal structure of n-eicosane. With both thermal conductivity and phase change capability considered, CPF3.5 was selected for further discussion.

**Fig. 4.**
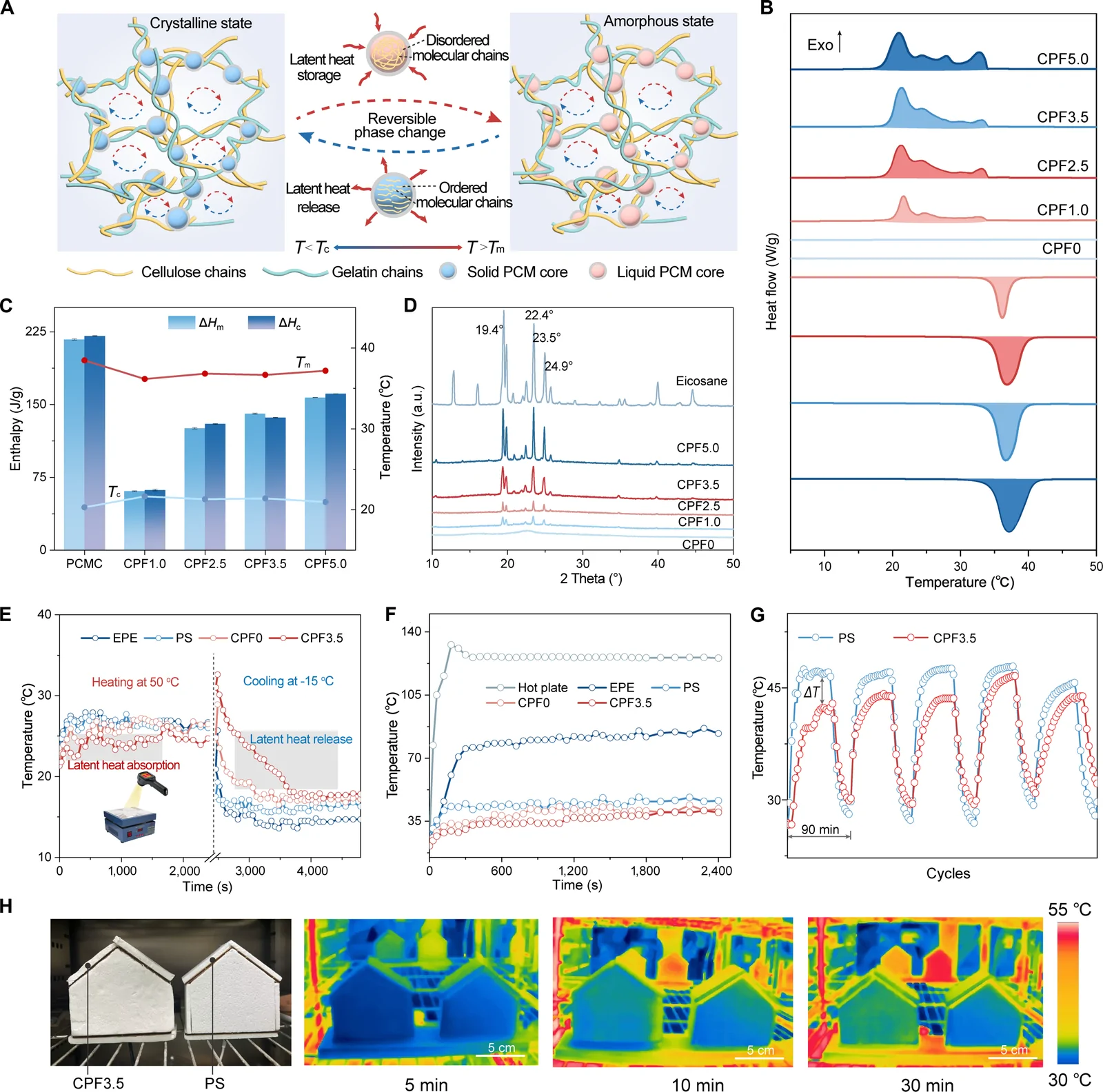
Thermal regulation performance and thermal stability of CPF. (A) Thermal regulation mechanism of CPF. (B) DSC curves of CPF with different microcapsule contents. (C) Enthalpy and phase change temperature of CPF with different microcapsule contents. (D) XRD patterns of *n*-eicosane and CPF with different microcapsule contents. (E) Time–temperature curves of CPF0, CPF3.5, EPE, and PS foams during heating at 50 °C and cooling at −15 °C. (F) Time–temperature curves of CPF0, CPF3.5, EPE, and PS foams at 120 °C. (G) Temperature profile inside the architectural model during an alternating cycle of 50 and 28 °C. (H) Optical and thermal infrared images of the architectural model after being heated at 50 °C for 30 min.

To confirm the thermal regulation capability of CPF3.5, we conducted thermal and cold shock tests. CPF0, CPF3.5, EPE, and PS foams with identical dimensions were placed on an isothermal heating stage at 50 °C and a cooling stage at −15 °C, and their temperature changes were monitored using a thermal infrared camera (Fig. 4E and Fig. S13). CPF0, EPE, and PS foams, which are conventional thermal insulation materials without PCM, exhibited a faster rise in surface temperature during heating until reaching thermal equilibrium. In contrast, CPF3.5 maintained the lowest heating rate throughout the 40-min testing period, with its surface temperature remaining stable at approximately 25 °C. During the cooling stage, benefiting from the crystallization exothermic effect of PCM, the temperature decrease of CPF3.5 was considerably slowed. The time required for CPF3.5 to cool down to a given temperature was more than twice as long as that for CPF0, EPE, and PS foams. This synergistic mechanism, combining thermal insulation with endothermic/exothermic buffering, endows the CPF with exceptional dynamic temperature-regulating performance and robust resistance to thermal/cold shocks. To evaluate the thermal reliability of CPF during heating and cooling processes, 50 successive temperature cycling tests were conducted within the temperature range of 5 to 60 °C (Fig. S14). The results demonstrated that the endothermic and exothermic curves of CPF3.5 remain essentially unchanged, confirming its excellent cycling stability.

Poor thermal stability is a critical issue for traditional commercial foams used in practical applications. To evaluate the stability of CPF3.5 in high-temperature environments, the leakage behavior of its phase change material was first tested. After heating at 60 °C for 24 h, CPF3.5 exhibited a leakage rate of only 1.0 ± 0.3% (Fig. S15), demonstrating its excellent leakage resistance. Subsequently, CPF0, CPF3.5, and commercial EPE and PS foams were placed on a thermostatic hot plate maintained at 120 °C, while their surface temperatures were monitored in real time using an infrared thermal camera (Fig. 4F and Fig. S16). The surface temperatures of both the EPE and PS foams rapidly increased. The EPE foam melted and underwent structural collapse, while the PS foam exhibited pronounced thermal deformation at its base. In contrast, after heating at 120 °C for 40 min, both CPF0 and CPF3.5 maintained a stable surface temperature of approximately 40 °C while preserving their structural integrity and stability. These results demonstrate that CPF3.5 combines excellent leakage resistance with robust thermal stability, highlighting its potential for reliable thermal management under high-temperature conditions.

To further evaluate the thermal regulation performance of CPF3.5, CPF3.5 and PS foam were used as insulation layers and applied to the surface of building model constructed with wooden substrates (Fig. 4H). The building model was placed in an oven at 50 °C and heated for 1 h; it was then removed and allowed to cool at room temperature (≈28 °C) for 30 min. This process was repeated for 5 cycles, and temperature changes inside the model were continuously recorded (Fig. 4G). During the initial heating phase (the first 10 min), the rate of temperature increase in CPF3.5-covered model was comparable to that of the PS foam-covered model. However, as heating progressed, the PCMCs gradually melted and absorbed heat, thereby slowing down the rate of temperature rise inside the hut and resulting in a final equilibrium temperature noticeably lower than that of the PS foam group. Upon transferring the model to indoor conditions, it exhibited similar temperature-response characteristics to those observed under heating conditions. Furthermore, the changes in the outer surface temperature of the hut during the 30-min heating process were recorded using a thermal infrared camera, revealing a lower surface temperature for CPF3.5-covered hut (Fig. 4H). These results fully confirm that the reversible phase transition of PCMCs within CPF3.5 can effectively absorb and release heat, effectively suppressing temperature fluctuations, thereby endowing the material with excellent thermal buffering capacity. This provides strong support for its practical application in the field of building thermal management.

### Hydrophobicity, sound absorption, closed-loop recyclability, and biodegradability

The abundant active hydroxyl groups on cellulose molecular chains endow the foam with strong hydrophilicity, rendering it prone to structural softening or collapse in humid environments due to moisture uptake. To improve the long-term stability of the CPF, we applied a hydrophobic surface treatment via chemical vapor deposition by grafting polydimethylsiloxane (PDMS), which effectively lowered the surface energy and raised the water contact angle to 124° (Fig. S17). This markedly suppressed water intrusion, transforming the foam from hydrophilic to hydrophobic, and thereby enhancing its stability.

Sound absorption is a critical function of porous materials. Owing to the lightweight and highly porous characteristics of CPF3.5, its sound absorption performance was evaluated using the transfer-function method. As shown in Fig. 5A, CPF3.5 with a thickness of 15 mm exhibited excellent broadband sound absorption performance in the 100 to 6,300 Hz frequency range. The sound absorption coefficient (SAC) generally increased with increasing frequency in the low-to-middle frequency range, reaching a maximum value of approximately 0.95 at around 2,000 Hz, followed by a gradual decrease. Nevertheless, CPF3.5 maintained a relatively high SAC in the high-frequency region, with an average SAC of 0.83 over 2,000 to 6,300 Hz. The NRC was further employed to quantitatively evaluate the sound absorption performance of the material [50]. CPF3.5 exhibited an NRC of 0.54, outperforming several previously reported sound-absorbing materials (Fig. 5B). The broadband absorption primarily originates from multiscale acoustic-energy dissipation within the hierarchical porous architecture. Acoustic waves undergo repeated reflection, scattering, and viscous friction within the interconnected pore walls and fibrous network, promoting the conversion of acoustic energy into heat (Fig. 5C). Meanwhile, SEM images (Fig. 2D) reveal numerous protruding PCMCs embedded along the plateau borders. These rough and irregular interfaces increase acoustic scattering and solid–acoustic interactions, thereby enhancing interfacial frictional dissipation and further improving the overall sound absorption performance. As a proof-of-concept demonstration for noise reduction, we evaluated the sound absorption performance of CPF3.5. As shown in Fig. 5D, a buzzer generated 102.2 dB of noise, which was reduced by 12.2 and 9.5 dB after passing through commercial EPE and PS foams, respectively. In contrast, CPF3.5 attenuated the noise by 23.0 dB, outperforming the commercial foams. These results demonstrate the potential of CPF3.5 for broadband noise-control and acoustic-protection applications.

**Fig. 5.**
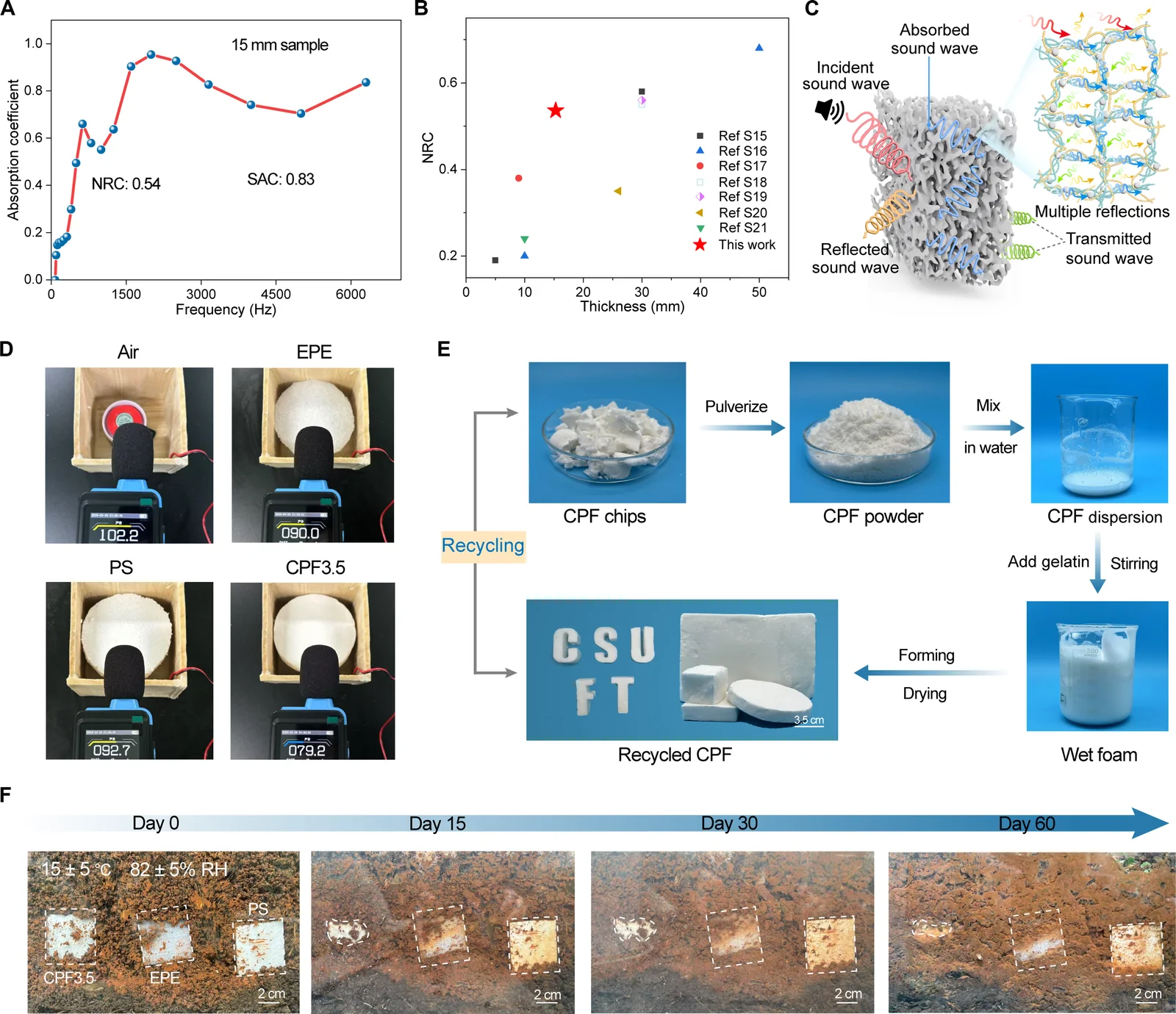
(A) SAC of CPF3.5 at a thickness of 15 mm. (B) Comparison of CPF3.5 with various reported sound-absorbing materials of different thicknesses in terms of NRC. (C) Sound absorption mechanism of CPF3.5 (relevant references are provided in the Supplementary Materials). (D) Photograph of CPF3.5 being used to provide noise insulation from a buzzer. (E) Illustration of the recycling process of CPF3.5. (F) Images of the biodegradation test of CPF3.5, EPE, and PS foams.

Recycling is a crucial component of the resource cycle and also an effective pathway to industrial decarbonization. The recyclability of CPF primarily stems from the hydrogen-bonding and physical entanglement network formed among cellulose, gelatin, and PCMCs, which enables the material to redisperse in an aqueous medium and undergo structural reconstruction. To further demonstrate the closed-loop recycling potential of CPF, we investigated its recycling and reuse process (Fig. 5E). First, waste CPFs were pulverized, dispersed in water, and subjected to high-speed shearing, followed by the addition of a small amount of gelatin to reform the wet foam. The resulting wet foam was then molded and freeze-dried to obtain recycled CPF. The recycled foam exhibited a low density of 0.035 g/cm^3^ while retaining excellent temperature-regulation performance (Fig. S18) and formability. SEM observations (Fig. S19) revealed that the recycled foam retained a hierarchical porous network composed of cellulose, gelatin, and PCMCs that was similar to that of the pristine foam, further demonstrating the critical roles of the reversible physical network and gelatin-mediated interfacial anchoring in enabling closed-loop recycling.

Unlike most polymers with high molecular weights, lignocellulose and gelatin are common nutrients for many microorganisms [51]. To evaluate the biodegradability of CPF3.5, samples of CPF3.5, commercial EPE, and PS foams with identical dimensions were buried in a custom-built degradation apparatus, and their morphological evolution was monitored over time (Fig. 5F). After 60 d of soil burial, CPF3.5 exhibited severe structural collapse accompanied by a substantial reduction in volume. SEM and FT-IR analyses of the residual samples confirmed that the gelatin and prat of the cellulose within CPF3.5 underwent extensive degradation, whereas the microcapsules remained structurally intact (Figs. S20 and S21). This phenomenon is attributed to the biological digestion of the cellulose and gelatin in CPF3.5 foam by specific extracellular enzymes (such as cellulases and proteases) secreted by soil microorganisms. The biodegradation process converts these polymers into small-molecule substances, ultimately leading to the physical collapse and decomposition of the foam skeleton. Furthermore, the PCMCs retained their intact spherical structure and stability, indicating that they can be recovered and reused through a simple washing process. This strategy not only prevents PCMCs from lingering in the environment as microplastics but also achieves the closed-loop recycling of high-value PCM. In contrast, EPE and PS foams remained unchanged after the same burial period, indicating the adverse environmental impact of nondegradable foams.

## Conclusion

This study reported a temperature-adaptive CPF produced via gelatin-mediated interfacial engineering. The structure of the CPF incorporated gelatin to facilitate interfacial assembly and structural reinforcement. Through thermally reversible gelation, gelatin induced a molecular chain transition into a triple-helix structure below the gel point, locking moisture within the wet foam and inhibiting bubble coalescence and drainage. During this process, gelatin acted as a surface-active component attached to the surface of the PCMCs. It also formed a hydrogen-bonding network and physical entanglements with cellulose after drying, which anchored the PCMCs within the plateau borders. This achieved functional integration and structural reinforcement of CPF. The as-prepared CPF exhibited a low density (0.044 g/cm^3^), low thermal conductivity [0.039 W/(m·K)], thermal insulation performance comparable to that of commercial foams, and good mechanical support properties. The phase-change capability of the PCMCs endowed the CPF with excellent thermal regulation performance, and it also demonstrated thermal stability at 120 °C. This strategy of precisely integrating functional components into a biomass skeleton broadens the potential applications of biomass foams and provides a new solution for the sustainable development of building thermal management and advanced thermal protection systems.

## Materials and Methods

### Materials

Poplar wood powder was sourced from Lingshou Chenyue New Material Co. Ltd. (Hebei Province, China). Hydrogen peroxide (H_2_O_2_, 30%), formic acid (HCOOH, 88%), sulfuric acid (H_2_SO_4_, >98%), PVA (1200), SDS, EA, and gelatin were purchased from Sinopharm Chemical Reagents Co. Ltd. PDMS, ammonium bicarbonate (AB), and EA were purchased from Macklin. Polyurea/*n*-eicosane PCMCs were purchased from Hebei Ruosen Technology Co. Ltd. Deionized water was prepared in the laboratory.

### Fabrication of cellulose

Poplar wood powder (80 to 100 mesh) was immersed in a formoperoxy acid solution and reacted at 50 °C for 6 h to remove lignin and hemicellulose. A solution of H_2_O_2_ and HCOOH at a molar ratio of 1:1 with 0.5 wt % H_2_SO_4_ (relative to the total mass of the solution) was used as a catalyst. Finally, the samples were washed with deionized water to neutrality and dried in an oven for later use.

### Fabrication of CPF

Cellulose suspension (4 wt %) was prepared by mixing cellulose (2 g) with deionized water (48 g) and then treated with a high-speed shear mixer at 10,000 rpm for 5 min to obtain a cellulose dispersion. The cellulose dispersion, 5 g of EA, and different mass ratios of PCMC (mass ratios to cellulose: 1:0, 1:1, 1:2.5, 1:3.5, and 1:5) were mixed uniformly. Subsequently, 10 g of gelatin solution (12 wt %) was added, and the mixture was stirred at 1,500 rpm for 15 min using a 4-blade impeller to form a wet foam. The wet foam was poured into a mold and frozen at −50 °C for 5 h, and the CPF was obtained by freeze-drying. The freeze-dried samples were labeled as CPFX, where X represents the mass ratio of PCMCs (e.g., CPF3.5 corresponds to a cellulose-to-microcapsule mass ratio of 1:3.5). To investigate the stability of CPF, comparative experiments were conducted using PVA and SDS as reference foaming agents. In these control groups, the cellulose concentration was kept constant, while the concentrations of PVA and SDS were set equal to that of gelatin in the CPF. All samples were subjected to mechanical stirring at 1,500 rpm for 15 min to generate the wet foams.

### Hydrophobic treatment of CPF

The hydrophobic treatment of CPF was carried out by a vapor deposition method. The cellulose/PCMC phase change foam was placed with PDMS and AB (*m*PDMS:*m*AB = 1:2) in a sealed desiccator, which was then placed in an oven at 80 °C for 6 h to allow the deposition reaction to proceed.

## Data Availability

All data needed to evaluate the conclusions in the paper are present in the paper and the Supplementary Materials.

## Supplementary Materials

Supplementary 1 Note S1 Figs. S1 to S21 Table S1
