# Supplementary material for: Gelatin-Mediated Interfacial Engineering of Cellulose-Based Phase Change Foams for Temperature-Adaptive Thermal Regulation
Source: Research (Wash D C). 2026 Nov 4;9:1473. doi: 10.34133/research.1473 (PMC13731818; doi:10.34133/research.1473)
Supplement: Supplementary file 1 [file research.1473.f1.docx]

**Supplemental Material**

**Gelatin-Mediated Interfacial Engineering of Cellulose-Based Phase Change Foams for Temperature-Adaptive Thermal Regulation**

Ya Zhou, Yingfeng Zuo^*^, Huan Gu, Ping Li, Xiaolong Guo, and Yiqiang Wu^*^

State Key Laboratory of Utilization of Woody Oil Resource, School of Materials and Energy, Central South University of Forestry and Technology, Changsha, Hunan, China

* Corresponding to:

zuoyf1986@163.com;

wuyiqiang@csuft.edu.cn;

**Note S1. Characterization methods.**

The morphology of wet foams was observed and imaged using an optical stereo microscope (AO-HK830-587, Shenzhen Aosvi Optical Instrument Co., Ltd., China), and the bubble stability was analyzed. The microstructure of the samples was characterized by scanning electron microscopy (SEM, MIRA3 LMH, TESCAN, Czech Republic) coupled with an energy-dispersive X-ray spectrometer (EDS, One Max 20, Oxford, UK). Attenuated total reflectance Fourier transform infrared spectroscopy (ATR-FTIR) was performed using a Nicolet iS50 (Thermo Fisher Scientific, USA) in the range of 4000-400 cm⁻¹ with a resolution of 4 cm⁻¹ and 32 scans. The obtained spectra were subjected to ATR correction, baseline correction, and normalization. X-ray diffraction (XRD) patterns of the samples were recorded using a D8 Advance A25 diffractometer (Bruker, USA) with Cu Kα radiation (λ = 1.541 Å) at 40 kV and 100 mA over a 2θ range of 10°-80°. Two-dimensional (2D) small angle X-ray scattering (SAXS) measurements were conducted using a Xeuss 2.0 system (Xenocs, France) equipped with Cu-Kα X-ray radiation of 0.154 nm wavelength. The phase transition behavior of the samples was investigated using differential scanning calorimetry (DSC, TA250, TA Instruments, USA). Micro-computed tomography (Micro-CT) was conducted using a Poincare 160 system (WTPI, China) at 40 kV and 200 µA with a spatial resolution of 5 µm. Based on the acquired cross-sectional images, three-dimensional reconstruction and porosity analysis were performed. Samples (35 × 35 × 35 mm^3^) were placed on heating stages at 50 °C and 120 °C, as well as on a cooling stage at −15 °C. An infrared thermal imaging camera (HM-TPK20-3AQF/W, HIKMICRO, China) was used to record temperature changes to evaluate the thermal insulation performance and thermal stability of the foams. The thermal conductivity of the foams at 25 °C and 50 °C was measured using a Hot Disk TPS2500S analyzer (Hot Disk AB, Sweden). The sound absorption performance was evaluated using an impedance tube system (SW 422 and SW 477, Beijing Shengwang Sound and Electric Technology Co., Ltd., China). Large (100 mm diameter) and small (30 mm diameter) tubes were used to measure the sound absorption coefficients (SAC) in the frequency ranges of 60-1800 Hz and 1600-6300 Hz, respectively. The noise reduction coefficient (NRC) was calculated as the arithmetic mean of the sound absorption coefficients at 250, 500, 1000, and 2000 Hz.

*Bulk density:* Foam samples with dimensions of 35 × 35 × 35 mm^3^ were prepared using a mold. The mass was recorded, and the volume was calculated. The density of the samples was determined using the equation (1). Each group of samples was measured three times, and the average value was calculated to ensure accuracy.

*ρ* = m/v	(1)

The porosity of CPF was calculated by:

$\mathrm{Porosity}=\left( 1-\frac{\rho}{\rho_{0}} \right)\times100\%$	(2)

Where *ρ* is the bulk density of the CPF sample, and *ρ*_0_ is the density of the solid skeleton estimated from the mass fractions of cellulose, gelatin, and PCMCs. The densities of cellulose (1.5g/cm^3^) and gelatin (1.35g/cm^3^) were obtained from the Ref.S22 and Ref.S23, respectively. Whereas the density of PCMCs (0.97g/cm^3^) was measured using a gas pycnometer (AccuPyc II 1340, Micromeritics, USA). For CPF3.5, the mass fractions of cellulose, gelatin, and PCMCs were 19.6%, 11.8%, and 68.6%, respectively.

*Theory Calculation*: In Materials Studio 2023, cellulose, gelatin, and polyurea molecules were constructed and geometrically optimized using the DFT method of the Dmol3 module. The exchange‑correlation functional was described by the generalized gradient approximation (GGA) with BLYP parameterization. The convergence tolerances for energy were set to 1×10^-5^ Ha/Å. The optimized geometries of the molecules were utilized to calculate the binding energy (E_B_) by the following equation:

E_B_ = E_total_– E_1_ – E_2_	(3)

where E_total_ represents the total energy of the molecular composite, and E_1_ and E_2_ are the energies of molecule 1 and molecule 2, respectively.

*The leakage-rate test.* Each sample was weighed and placed on filter paper, then put in an oven at 60 °C. At regular intervals the specimen was reweighed to record its mass, and the filter paper was replaced. The leakage rate (W, %) was calculated using the following equation:

W =(M_0_−M_1_)/M_0_×100	(4)

Where M_0_ is the initial mass of CPF3.5 and M_1_ is the mass of CPF3.5 after the leakage test.

*Biodegradation test:* To facilitate observation of the biodegradability of CPF3.5, EPE, and PS foam materials, soil was collected from an undisturbed area and placed in a glass container. Samples with dimensions of 40 × 40 × 5 mm^3^ were buried at a depth of 60 mm in the soil, and their morphological changes were recorded using digital photography.

*Mechanical properties tests:* The compressive and three-point bending test of CPF was evaluated using a universal testing machine (AGX-10kNV2D, SHIMADZU, Japan). Test specimens with dimensions of 20 × 20 × 20 mm^3^ were prepared for compressive testing. The dimensions of the specimens for the three-point bending test were 30 × 15 × 6 mm^3^, with a test span of 18 mm. A constant loading rate of 0.5 mm/min was applied for both three-point bending and compressive tests. To ensure the reliability of the results, at least three parallel tests were performed for each test sample.

*Rheological measurement*：The rheological properties of the wet foams were measured at 20 °C using a rotational rheometer (DHR-2, Waters, USA). A 40 mm diameter parallel plate geometry was used, with a gap set at 500 μm. Prior to testing, the samples were equilibrated at 20 °C for 2 min. To determine the linear viscoelastic region (LVR), an amplitude sweep was performed at a fixed frequency of 10 rad/s over a strain range of 0.01–10%. Based on these results, a strain value of 0.4% was selected for subsequent rheological tests. Finally, the storage modulus (G') and loss modulus (G'') of the wet foams were measured as a function of oscillation frequency ranging from 0.01 to 10 Hz.

**Figure S1.** Stability of the wet foam. (a) Viscosity of wet foams prepared with different foaming agents. (b) Stability of wet foams prepared with different foaming agents after 30 min.

A quasi-2D confinement method based on the Hele-Shaw cell principle was employed to directly observe the morphology and temporal evolution of the wet foam, aiming to evaluate its stability. Briefly, a micro-thin-layer chamber with a controlled gap was constructed by placing two cover slips (24 × 24 × 0.17 mm³) as spacers between two parallel glass slides (25.4 × 76.2 × 1 mm³), which were secured at both ends with binder clips. The prepared wet foam was uniformly injected along the edge of the chamber using a dropper, filling the gap via capillary action or gentle compression. After cleaning the exterior surfaces, the cell was placed under an optical microscope for in-situ observation. This setup allowed for a precise assessment of foamability and coarsening behavior within a constrained environment.

**Figure S2.** Time-dependent micromorphological evolution of CPF wet foams with varying PCMC loading contents.

**Figure S3.** Optical microscopy image of CPF0 and CPF3.5 placed at room temperature for 36 h.

**Figure S4.** SEM image of CPF1.0, CPF2.5 and CPF5.0 at different magnifications.

**Figure S5.** EDS elemental mapping images of (a) PCMC, (b) CPF0, and (c) CPF3.5.

**Figure S6.** FT-IR spectra of the CPF foam with varying PCMC contents.

**Figure S7.** Secondary structure content of proteins CPF0 and CPF3.5.

**Figure S8.** (a) Three-dimensional structure of CPF3.5 based on Micro-CT analysis. Micro-CT image of (b) yz-plane, (c) xz-plane, and (d) xy-plane.

**Figure S9.** Pore size and porosity analysis of CPF3.5 based on Micro-CT characterization.

**Figure S10.** Digital photos during the three-point bending test of CPF3.5.

**Figure S11.** Temperature rise curves of CPF0 and CPF3.5 during thermal conductivity testing at 25 °C.

**Figure S12.** DSC curves of n-eicosane and PCMC.

**Figure S13.** (a) Temperature profiles of EPE, PS, CPF0 and CPF3.5 during the heating and (b) the cooling process.

**Figure S14.** DSC curve of CPF after 50 heating-cooling cycles.

**Figure S15.** Leakage curves of CPF3.5.

**Figure S16.** Comparison of the bottom morphologies of CPF0, CPF3.5, EPE, and PS foams after testing on a 120 °C heating platform.

**Figure S17.** Hydrophobic properties of CPF3.5 modified by vapor deposition. (a) Evolution of static water contact angle over time (0-300 s). (b) Digital image of dyed water droplets on the CPF surface. (c) Digital photo of CPF3.5 dripping dynamically.

**Figure S18.** Thermal performance of recycled CPF. (a) Time-temperature curves of recycled CPF during heating at 50 °C. (b) Thermal insulation properties of recycled CPF.

**Figure S19.** Microscopic images of recycled CPF. (a) SEM images of recycled foam at different magnifications. (b) EDS elemental mapping images of recycled CPF.

**Figure S20.** SEM and EDS elemental mapping images of CPF3.5 after 60-day soil burial.

**Figure S21.** FT-IR spectra of CPF3.5 after 60-day soil burial.

**Table S1**. Comparison of energy storage properties and thermal conductivity with those of the corresponding references.

| **Supporting Materials** | | **T_m_/T_c_ (℃)** | **ΔH (J/g)** | **Thermal Conductivity（ W/(m·K)）** | **Refs** |
| --- | --- | --- | --- | --- | --- |
|  | CNT/MXene | 12.5;48.6 | 36.8 | 0.15 | Ref.S1 |
|  | PLA aerogel | 60.62;70.31 | 199.3 | / | Ref.S2 |
|  | Cellulose/PEG | -5.4;7.7 | 78.6 | / | Ref.S3 |
|  | Cellulose/PEG foam | 36.9;58.3 | 130 | 0.35 | Ref.S4 |
|  | BTC foam | 62.0;53.1 | 171.7 | 0.08 | Ref.S5 |
|  | CNF/PCMC aerogel | 28.4;41.1 | 174.9 | 0.0443 | Ref.S6 |
|  | PVA/PEG/CNC aerogel | 32.0; | 44.18 | 0.0423 | Ref.S7 |
|  | WPU/PCMC/BaSO_4_ | 19.6;28.6 | 150 | 0.063/0.197 | Ref.S8 |
|  | EVA/PW/MWCNTs | 53 | 116.5 | / | Ref.S9 |
|  | CMA4 | 26.4 | 146.1 | 0.040 | Ref.S10 |
|  | CMP | 40.0~50.7 | 68.0~152.1 | 0.041~0.082 | Ref.S11 |
|  | PS aerogel | 23.38;22.53 | 60.7 | 0.029 | Ref.S12 |
|  | PCC aerogel | 25.0 | 52.8 | / | Ref.S13 |
|  | SA/MXene/PEG | 60.66 | 142.3 | / | Ref.S14 |
|  | CPF0 | / | 0 | 0.034 | This work |
|  | CPF1.0 | 36.15;21.66 | 60.8 | 0.036 |  |
|  | CPF2.5 | 36.83;21.30 | 125.6 | 0.040 |  |
|  | CPF3.5 | 36.67;21.4 | 140.7 | 0.039 |  |
|  | CPF5.0 | 37.1720.95 | 157.3 | 0.046 |  |

**References**

[S1] Han L, Luo T, Kang H, Liu G, Fang Q. Bio-based, phase-change MXene/CNT foams for integrated electromagnetic interference shielding, thermal management and infrared stealth. *Adv. Nanocomposites*. 2025; 2: 148-161.

[S2] Yin G, Yang X, Lópe A, Ao X, Wang M, Molleja J, Wang D. PLA aerogel as a universal support for the typical organic phase change energy storage materials. *J. Energy Storage*. 2023; 73:108869.

[S3] Gök Ö, Alkan C, Konuklu Y. Developing a poly(ethylene glycol)/cellulose phase change reactive composite for cooling application. *Sol. Energy Mater. Sol. Cells*. 2019; 191:345-349.

[S4] Baniasadi H, Ziba Fathi, Abidnejad R, Silv P, Bordoloi S, Vapaavuori J, Niskanen J, Lizundia E, Kontturi E, Lipponen J. Biochar-infused cellulose foams with PEG-based phase change materials for enhanced thermal energy storage and photothermal performance. *Carbohydr. Polym*. 2025; 367:123999.

[S5] Huan S, Wang H, Yang Z, Qiao L, Li C, Wang C, Huan S. All-green foam for thermal management via pickering templating. *Bioresour. Technol.* 2026; 441:133638.

[S6] Li S, Cao H, Liu W, Bai L, Wang Y, Yang J, Yang W. Ambient-dried phase-change functionalized thermal insulation materials with latent heat charging/discharging capacities and superior temperature-adaptive thermal regulation performance. *Adv Funct Mater.* 2026;36(8):e16888.

[S7] Wang F, Zhou J, Qiao L, Hu S, Ji X, Sun X, Sun M, Liu Y, Li Y, Yao T, Zhu J, Ma Q, Gu Y, Cui S, Yang H, Wang C. Bioinspired biodegradable sandwich-structured porous metafabric for passive personal thermal management. *Adv Fiber Mater.* 2025;7:1844-1858.

[S8] W. Wei, Y. Tian, Z. Shao, Bao B, Chen G, Chen D, Wu C, Wu B, Chen J, Hou C, Wang H. Self-Adaptive Metafabric Enabling Thermal Rectification and Radiative–Phase Change Energy Cycling for All-Weather Thermal Regulation. *Adv. Funct. Mater.* 2026; 36(11): e15535.

[S9] Chen J, Tang Z, Zou Y, Zhang G, Qi X, Wang Y. Mass-produced multifunctional composite foams based on an electrical conductivity-phase change-shape memory integrated skeleton toward electromagnetic interference shielding and infrared stealth applications. *Polym.* 2026; 359(15):130262.

[S10] Xiong F, Jin Y, Han H, Chu H, Jia K, Qin M, Wan Y, Usman A, Geng X, Zhong R, Shen Z, Zou R. Phase change aerogels enabled with passive radiative cooling: toward intelligent thermal management. *Adv. Funct. Mater.* 2026; 36(50): e76004.

[S11] Xiong F, Zhou J, Jin Y, Zhang Z, Qin M, Han H, Shen Z, Han S, Geng X, Jia K, Zou R. Thermal shock protection with scalable heat-absorbing aerogels. *Nat Commun.* 2024; 15:7125.

[S12] Guo J, Zhu L, Ba X, Li X, Feng Y, Li S, You R. Phase change microcapsule-functionalized silk nanofibrous aerogel for thermal management. *ACS Appl. Polym. Mater*. 2025; 7 (17): 11818–11826.

[S13] Li J, Zhang Y, Zhang Z, Liu H, Ru J, Li Y, Chen Z. Preparation of phase change microcapsules-aerogels composites and the enhanced thermal properties. *Mater. Lett.* 2020; 268:127563.

[S14] Niu C, Li M, Pan G, Lu J, Yu H, Ge X, Feng W. Sandwich-structured porous foam from sodium alginate-intercalated MXene/polyethylene glycol: Photothermal conversion, phase change storage and electromagnetic interference shielding. *Carbohydr. Polym.* 2026; 381:125143.

[S15] Pang K, Liu X, Pang J, Samy A, Xie J, Liu Y, Peng L, Xu Z, Gao C. Highly Efficient Cellular Acoustic Absorber of Graphene Ultrathin Drums. *Adv Mater.* 2022;34(14):2103740.
[S16] Zong D, Bai W, Yin X, Yu J, Zhang S, Ding B. Gradient Pore Structured Elastic Ceramic Nanofiber Aerogels with Cellulose Nanonets for Noise Absorption. *Adv Funct Mater.* 2023;33(31):2301870.
[S17] Wang J, Huang C, Chen J, Zhou Z, Xu D, He C, Chen C, Li X. A Sustainable Route toward Gradient-Structured Sponge with Topological Cellular Cavity from Bamboo for All-Season Comfort. *Adv Funct Mater.* 2025; article e32182.
[S18] Yu C, Shi Q, Zhao H, Guo J, Lin D, Yao Y, Zhang X, Jiang X. Multilayer Polyvinyl Alcohol/Carbon Composite Aerogels with Broadband Microwave and Noise Absorption and Novel Shape Memory Effect. *Adv Funct Mater.* 2025;35(43):2502749.
[S19] Zong D, Cao L, Yin X, Si Y, Zhang S, Yu J, Ding B. Flexible ceramic nanofibrous sponges with hierarchically entangled graphene networks enable noise absorption. *Nat Commun.* 2021;12(1):6599.
[S20] Nine M J, Ayub M, Zander A C, Tran D N H, Cazzolato B S, Losic D. Graphene Oxide-Based Lamella Network for Enhanced Sound Absorption. *Adv Funct Mater.* 2017;27(46):1703820.
[S21] Shen S, Jiang Z, Lu Y, Feng K, Wu Y, Zhou K, Zhang D, Zhang Y. Elastic aerogels prepared by cyclic freeze casting for broadband sound absorption and sleep health monitoring. *Nano Energy.* 2026;147:111591.

[S22] Frka-Petesic B, Parton T, Honorato-Rios C, Narkevicius A, Ballu K, Shen Q, Lu Z, Ogawa Y, Haataja J, Drogue B, Parke R, Vignolini S. Structural Color from Cellulose Nanocrystals or Chitin Nanocrystals: Self-Assembly, Optics, and Applications. *Chem. Rev*. 2023; 123(23): 12595-12756.

[S23] Laktionova A, Dobrynina E, Zykova V, Surovtsev N. Brillouin Scattering Study of Gelatin Films with Different Water Concentrations. *Bull. Russ. Acad. Sci. Phys*. 2023; 87: 66-70.
